# Development and validation of the student ratings in clinical teaching scale in Australia: a methodological study

**DOI:** 10.3352/jeehp.2023.20.26

**Published:** 2023-09-05

**Authors:** Pin-Hsiang Huang, Anthony John O’Sullivan, Boaz Shulruf

**Affiliations:** 1 Department of Medical Humanities and Education, College of Medicine, National Yang Ming Chiao Tung University, Taipei, Taiwan; 2 Office of Medical Education, Faculty of Medicine and Health, The University of New South Wales Sydney, Sydney, Australia; 3 Division of Infectious Disease, Department of Medicine, Taipei Veterans General Hospital, Taipei, Taiwan; 4 Faculty of Medicine and Health, The University of New South Wales Sydney, Sydney, Australia; 5 Department of Endocrinology, St George Hospital, Sydney, Australia; 6 Centre for Medical and Health Sciences Education, University of Auckland, Auckland, New Zealand; Hallym University, Korea

**Keywords:** Medical students, Reproducibility of results, Statistical factor analysis, Surveys and questionnaires, Australia

## Abstract

**Purpose:**

This study aimed to devise a valid measurement for assessing clinical students’ perceptions of teaching practices.

**Methods:**

A new tool was developed based on a meta-analysis encompassing effective clinical teaching-learning factors. Seventy-nine items were generated using a frequency (never to always) scale. The tool was applied to the University of New South Wales year 2, 3, and 6 medical students. Exploratory and confirmatory factor analysis (exploratory factor analysis [EFA] and confirmatory factor analysis [CFA], respectively) were conducted to establish the tool’s construct validity and goodness of fit, and Cronbach’s α was used for reliability.

**Results:**

In total, 352 students (44.2%) completed the questionnaire. The EFA identified student-centered learning, problem-solving learning, self-directed learning, and visual technology (reliability, 0.77 to 0.89). CFA showed acceptable goodness of fit (chi-square P<0.01, comparative fit index=0.930 and Tucker-Lewis index=0.917, root mean square error of approximation=0.069, standardized root mean square residual=0.06).

**Conclusion:**

The established tool—Student Ratings in Clinical Teaching (STRICT)—is a valid and reliable tool that demonstrates how students perceive clinical teaching efficacy. STRICT measures the frequency of teaching practices to mitigate the biases of acquiescence and social desirability. Clinical teachers may use the tool to adapt their teaching practices with more active learning activities and to utilize visual technology to facilitate clinical learning efficacy. Clinical educators may apply STRICT to assess how these teaching practices are implemented in current clinical settings.

## Graphical abstract

[Fig f3-jeehp-20-26]

## Introduction

### Background/rationale

Measuring clinical teaching efficacy relies on student ratings, and most universities use these to evaluate teaching. Student ratings are often sought at the completion of teaching activities using standardized rating forms. However, many clinical teaching measurements by student ratings fail to cover all aspects of clinical teaching methodology, and the instruments used often have limited evidence of validity [1]. Moreover, there is no consensus as to which practice is most effective. A recent meta-analysis investigated the effectiveness of teaching-learning factors (TLFs) in clinical education and provided a comprehensive overview of the relative effectiveness of different clinical teaching methods [2]. The resulting list of effective TLFs is now available for clinical educators as a useful source.

Commonly, evaluation scale anchors allow participants to report the extent to which they agree with each given statement (e.g., 5-point Likert scale from “strongly disagree” to “strongly agree”). However, agreement scales may reflect respondents’ attitudes towards behaviors instead of reporting their real experience or effectiveness of the behavior. Both “social desirability,” a tendency to present oneself positively, and “acquiescence,” a passive endorsement of an assertive statement despite the descriptions, may result in respondents answering with positive agreement because students may be polite, empathize with the teacher, and wish to avoid supplying a negative response [1]. Conversely, when applying frequency scales, respondents focus on the behaviors or incidents and recall how often they occur [3]. Frequency scales are considered minimally biased because they are less likely to assess respondents’ attitudes or the intensity of their perceptions [3]. Therefore, in comparison to agreement scales, frequency scales may minimize or alleviate “acquiescence” or “social desirability” effects.

Identifying effective clinical teaching methods and applying robust methods of tool development and evaluation provide the opportunity to develop a reliable and valid tool to measure clinical teaching practices [2].

### Objectives

The current study aims to appraise the reliability and validity of the newly introduced Student Ratings in Clinical Teaching (STRICT) scale used to evaluate clinical teaching practices.

## Methods

### Ethics statement

Ethics approval number HC180496 was applied to medical students studying at the University of New South Wales in 2018. Approval was granted by the University of New South Wales review panels (HREAPG: Health, Medical, Community and Social). Informed consent was obtained from participants before they answered to the questionnaire.

### Study design

This was a cross-sectional study for validating a questionnaire.

### Setting

The medicine program at the University of New South Wales, Sydney, Australia is 6 years in duration with approximately 280 students per year. Medical students in the second, third, fifth, and sixth years receive clinical training. Data were collected in 2018.

### Participants

The participants were recruited from undergraduate medical students in years 2, 3, and 6 studying at the Faculty of Medicine and Health, University of New South Wales in 2018, and 834 medical students were invited to participate. The number of students who responded to the questionnaire was 352 (42.2%), among whom 157 (44.6%) were men, 183 (52.0%) were women, and 3.4% provided no data on gender. The mean age was 22.34 years (standard deviation [SD]=1.94 years). No exclusion criteria were applied.

### Data sources/measurement

**Fig. 1.**
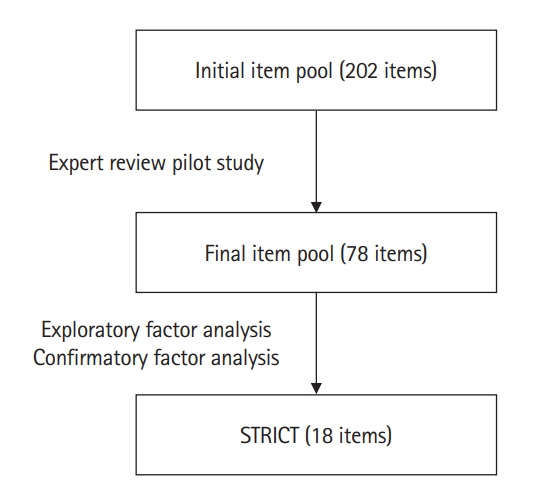
Flow chart of Student Ratings in Clinical Teaching (STRICT) development.

The development of STRICT was derived from a meta-analysis of the effectiveness of clinical teaching [2] and used the 16 effective TLFs identified to construct the scale. The listed TLFs included, but were not limited to, mastery learning, concept mapping, visual-perception programs, problem-solving teaching, interactive video methods, and student-centered teaching. The authors initially generated an item pool of 202 items representing all TLFs. The item pool was then reviewed by 3 experts (1 scale expert and 2 clinicians), followed by a pilot study (with medical administrators and fifth-year medical students) to ensure the contents were appropriate and unambiguous. This reduced the number of items from 202 to 78, and 6-point frequency scales from “never” (=1) to “always” (=6) were applied (Fig. 1).

### Variables

Demographic variables included the year of study, gender, and age. STRICT included 78 items (Supplement 1), and the latent variables were to be identified as part of the analysis.

### Bias

Due to the nature of a validation study, response bias might have existed, yet it is expected to be minimal and not detectable.

### Study size

The common recommended sample size is to recruit 10 participants per questionnaire item. It is also recommended that the sample size for performing exploratory factor analysis (EFA) and confirmatory factor analysis (CFA) of the questionnaire should be around 300, and the minimum size is 150 [4]. In this study, with an initial 78-item scale to be validated, a sample of 3 years of medical students (n=834) would be deemed acceptable if the response rate is >35%.

### Statistical methods

EFA was performed using the maximum likelihood method with oblimin rotation, and a factor loading of 0.45 was set as a cut-off point for items [5]. An eigenvalue of 1 was determined as the cut-off for an adequate amount of variance explained, and a scree plot was used to justify the cut-off point. CFA with structural equation modelling was performed using AMOS 24.0 (IBM Corp.), and correlations between factors, factor loading of each item, and model fit indices (chi-square, comparative fit index [CFI], Tucker-Lewis index [TLI], root mean square error of approximation [RMSEA], and standardized root mean square residual [SRMR]) were presented.

## Results

### Participants

Among 834 medical students invited to complete the questionnaire, 352 (44.2%) completed the questionnaire, 157 (44.6%), 183 (52%), and 8 (2.3%) were men, women, and unidentified, respectively. Of the respondents, 63 (17.6%), 134 (38.1%), and 151 (43.4%) were in years 2, 3, and 6 respectively; 4 (1.1%) did not report their year of study. The mean age was 22.34 (SD=1.84) with a range of 19–30 years.

### Main results

#### Exploratory factor analysis

After deleting the items with low factor loadings (<0.45), 18 of the 78 items remained, and 4 factors were identified through EFA. The factor loadings for each item and factor groupings are listed in Table 1, and the correlations between factors are shown in Table 2.

The first factor included items related to feedback and linkage from knowledge to practice (student-centered learning), and the second factor related to the utilization of visual technology (visual technology). The third factor included items relating to various problem-solving techniques (problem-solving learning), and the fourth dealt with self-reflection and goal setting (self-directed learning).

The reliability (Cronbach’s α) for each factor was 0.89, 0.85, 0.78, and 0.77, respectively; all were within the acceptable value of >0.7. In terms of factor correlation, a high positive correlation (r=0.59) was found between student-centered learning and self-directed learning, and a high negative correlation (-0.55) was found between student-centered learning and problem-solving learning. Visual technology had a low negative correlation with problem-solving learning (-0.28). Other factor correlations were within the moderate range (between |0.34| and |0.43|) (Table 2). However, the correlations are subject to change by adjusting delta in the oblimin rotation.

#### Confirmatory factor analysis

**Fig. 2.**
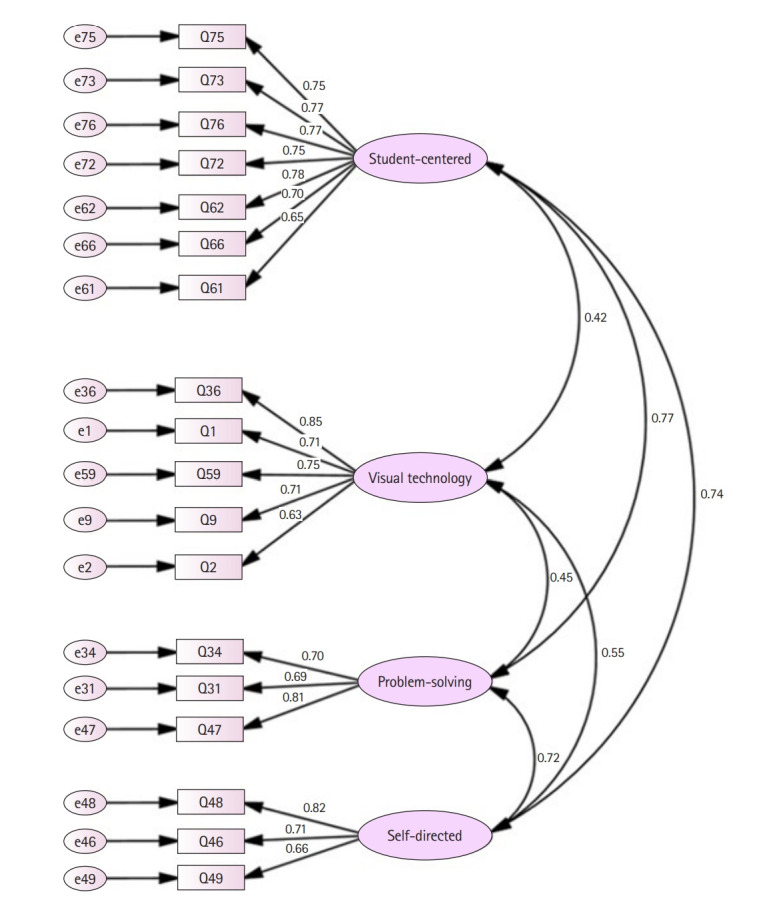
Structured model of Student Ratings in Clinical Teaching.

Using a first-order model, a further check with CFA was conducted (Fig. 2). The level of goodness of fit was acceptable for the chi-square test (*χ*^2^=345.71, degrees of freedom=129, P<0.01) and the CFI, as well as the TLI (also known as non-normed fit index) were also acceptable (CFI=0.930 and TLI=0.917) [6]. The RMSEA was 0.069, and the SRMR was 0.060; both were within the acceptable range.

The composite reliability was >0.7, which is within the acceptable to very good range (0.90 for student-centered learning, 0.85 for visual technology, 0.79 for problem-solving learning, and 0.78 for self-directed learning). It is noted however, that student-centered learning, problem-solving learning, and self-directed learning were highly correlated to each other (0.72 to 0.77), while visual technology had lower correlations with them (0.42 to 0.55) (Fig. 2). Overall, the results indicate that STRICT is a reliable measurement tool and its construct validity is supported by the statistical analysis results.

## Discussion

### Key results

The purpose of this study was to develop and validate an effective tool to evaluate clinical teaching practices reported by students. Four teaching practices were identified: student-centered learning, visual technology, problem-solving learning, and self-directed learning.

### Interpretation

Student-centered learning is often referred to as how students determine their learning goals and learning approaches with explicit guidance, as opposed to teacher-centered learning where teachers take control over learning goals, content, and progress. Seven items could be further converged into learning goals (no. 76, 72, 62), the teaching approach (no. 73, 66), and the peer-learning process (no. 75, 61). Student-centered learning is generally based on students’ autonomy to learn, combined with clear intended learning outcomes, supportive teaching approaches and cooperative peer learning [7]. These associations are also demonstrated in the higher correlations between the “student-centered,” “self-directed,” and “problem solving” factors (Table 2). In clinical education, students as well as practicing doctors are required to keep learning and updating their skills and knowledge, despite time-consuming clinical duties. Therefore, medical educators have advocated that learners should take responsibility for their own learning, and thus, over the past 2 decades, medical education reforms have shifted towards student-centered approaches [8]. Students are more self-motivated when the difficulties they encounter are recognized and supported by clinical teachers, and students’ stress is reduced throughout this process. Hence, medical students increasingly engage in more clinical learning, and these items could explain how they learn from peers, produce self-determined learning goals, and provide supportive and timely feedback.

Visual technology refers to the utilization of equipment to facilitate enhanced visual perception and learning experiences. Taking the visualization of the anatomical structure as an example, students may develop better understanding of anatomical and physiological interactions, enjoy the learning process more, and learn better if the learning is presented as a visual medium [9]. Out of 5 items loading on the visual technology factor, 3 items related to how technology enhances visual perception (no. 1, 36, 59), and 2 related to the activity (no. 2, 9). To illustrate how visual technology affects clinical learning, virtual reality, for example, provides students with opportunities to practice their skills and safely bridge the gap from knowledge to bedside practice [10]. In such environments, students are allowed to learn from errors without profound negative consequences and receive self-visual feedback through digital records in relevant computer-based simulations. Furthermore, clinical students will benefit from visual technology if it requires them to identify anatomical landmarks and structures, or interpret clinical images and laboratory data. In conclusion, the use of visual technologies helps students safely practice their knowledge and skills, and the interactive interface can enhance their spatial concepts of anatomy and physiological interactions, for example, enabling them to also practice decision-making, clinical skills, and reasoning.

Problem-solving learning focuses on students identifying, prioritizing, and solving problems with appropriate guidance and support from teachers. Three items loaded onto this factor, that is, problem identification, prioritization, and solution. In clinical settings, students practice analytical skills, such as blood test interpretation, and clinical reasoning through problem-solving learning. Although some criticism points towards the difficulty of integrating problem-solving learning in clinical settings, it is feasible to adopt this form of learning into daily tasks, such as dealing with certain disease manifestations [11]. Therefore, problem-solving learning may train students to efficiently identify problems and challenges in clinical practices and then divide the problems into manageable components; and with the aid of clinical reasoning, these approaches gradually facilitate students to learn and practice independently.

The traits of self-directed learning can be divided into task-oriented learning, student-teacher communication, and self-reflection. Three items loaded on self-directed learning and represent these 3 traits (no. 49 for task-oriented learning, no. 46 for student-teacher communication, and no. 48 for self-reflection). In clinical settings, students often learn by performing new tasks when they meet new problems. Therefore, this learn-from-tasks model is adapted to task-based learning, a component of problem-solving learning [12]. In addition, self-directed learning can be performed by a small group of students wishing to achieve certain goals or complete assessments, tasks and projects, and students in the same clinical attachment can learn independently whilst peer-teaching and cooperating; and this approach may result in them developing greater confidence as well as psychomotor and cognitive skills [13]. Moreover, it is important for clinical students to rethink what they have learnt and recognize what they still do not know, and how to improve and fill in knowledge and skills gaps. This process of reflection is referred to as using meta-cognitive skills and is often used in clinical reasoning. For example, Gibbs’ model of self-reflection explains how students make action plans based on reflecting upon past experiences, and students gradually improve their interviewing skills via this approach [14]. In conclusion, self-directed learning is closely related to problem-solving learning, small group learning and meta-cognitive strategies. However, the usefulness of these traits relies on how actively students initiate their learning. Students must take responsibility to set and meet their own goals and undertake frequent self-reflection. Therefore, these items reflect how students direct their learning and monitor their own progress; hence, they fit the term “self-directed learning” well.

STRICT’s main strengths are: (1) it is based on observation more than judgement which is preferable, particularly when used by non-experts; (2) it focuses on domains that are found most relevant for the quality of teaching [2]. STRICT’s main weakness is having only 3 items in 2 domains. Although acceptable, further research should aim to improve the STRICT tool by adding more items.

### Comparison with previous studies

In comparison to previous tools used to evaluate clinical teaching, STRICT successfully addresses some critical shortcomings of commonly used tools such as the Dundee Ready Education Environment Measure and Maastricht Clinical Teaching Questionnaire [15]. In particular, STRICT’s items were developed from robust meta-analysis that identified effective clinical teaching practices; the response anchors used a 6 point frequency scale, which is less vulnerable to bias; and the sample size used was diverse and large enough to include students from the first to final years in the medicine program. The findings of good reliability measures and model fit support the validity and robustness of STRICT.

### Limitations

A limitation of the study was that the sample size was not large enough to consider different teaching and learning conditions in different specialties. The student samples were collected from 11 hospitals and across more than 20 specialities; it was difficult, however, to undertake further analysis to include impacts of the various sites and specialities. Another limitation was that individual differences among students were not considered.

### Generalizability

The diversity of student experience, clinical setting, personal/demographic backgrounds, and the nature of the items that capture observable clinical teaching practices (rather than personal judgment) alongside the strong support of its validity as the psychometric indices suggest that STRICT is most likely to be a useful evaluation tool for clinical teaching globally. Nonetheless, further research is required to establish this by using additional empirical results from well-designed studies undertaken within diverse contexts.

### Suggestions

Future studies should look at the impact of individual differences across respondents and contexts. Validity and reliability should be checked across different countries and settings to understand its generalizability.

### Conclusion

STRICT is a valid and reliable tool that demonstrates how students perceive clinical teaching efficacy. A major difference of STRICT is that it measures the frequency of teaching practices rather than students’ judgement of them, and as such, it might mitigate the biases of acquiescence and social desirability. Based on these findings, clinical teachers might adapt their teaching practices to include more active learning activities and utilize visual technology to facilitate clinical learning efficacy. Clinical educators may apply STRICT to assess how these teaching practices are implemented in current clinical settings.

## Figures and Tables

**Figure f3-jeehp-20-26:**
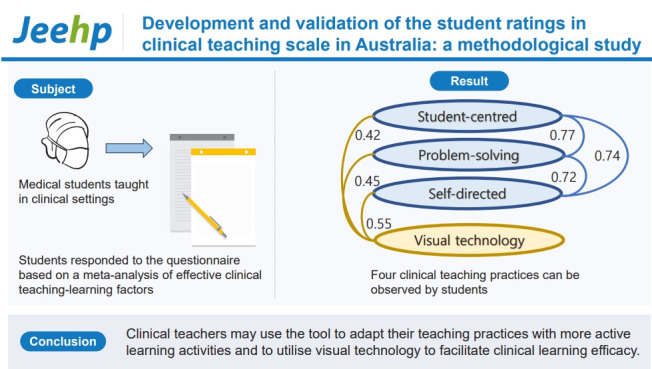


**Table 1.** Factor loadings of the items in a pattern matrix and a structure matrix

No.	Item	Pattern matrix				Structure matrix			
		Student-centered	Visual technology	Problem-solving	Self-directed	Student-centered	Visual technology	Problem-solving	Self-directed
75	The tutor facilitates students to learn from observing peers.	**0.81**	-0.01	-0.04	-0.11	**0.78**	0.23	-0.45	0.43
73	The tutor acknowledges students accomplishment during the teaching session.	**0.78**	-0.04	-0.04	-0.03	**0.77**	0.20	-0.46	0.55
76	The tutor explains the intended learning outcomes to students.	**0.77**	0.04	0.09	0.05	**0.77**	0.30	-0.37	0.49
72	The tutor links the teaching materials to the intended learning outcomes.	**0.68**	0.07	-0.11	-0.04	**0.77**	0.23	-0.44	0.38
62	The tutor addresses the gaps between individual students’ current performance and the intended learning outcomes.	**0.67**	-0.12	-0.06	0.19	**0.74**	0.32	-0.49	0.43
66	The tutor adapts their practice to address students' feedback on their teaching.	**0.58**	0.08	-0.01	0.12	**0.69**	0.33	-0.40	0.51
61	The tutor facilitates students to practice interpersonal communication skills with peers.	**0.57**	0.04	-0.03	0.09	**0.65**	0.28	-0.39	0.45
36	The tutor uses visual technology to enhance understanding of patterns.	0.03	**0.85**	-0.11	-0.10	0.33	**0.85**	-0.33	0.33
1	The tutor utilizes technology to illustrate learning.	-0.12	**0.75**	0.01	0.03	0.37	**0.76**	-0.15	0.42
59	The tutor uses technology to facilitates visual feedback to students.	0.17	**0.72**	0.16	0.08	0.15	**0.73**	-0.15	0.28
9	The tutor includes virtual activities in the learning materials.	-0.01	**0.68**	-0.02	0.03	0.25	**0.70**	-0.22	0.33
2	The tutor adds fun activities to the teaching session.	0.08	**0.46**	-0.17	0.12	0.41	**0.59**	-0.39	0.43
34	The tutor encourages students to consider a range of hypotheses in problem solving.	0.15	0.11	**-0.74**	-0.13	0.52	0.31	**-0.81**	0.28
31	The tutor prompts students to split a complex problem into its components.	0.05	0.02	**-0.54**	0.22	0.60	0.28	**-0.68**	0.59
47	The tutor demonstrates how to solve problems step by step.	0.14	-0.05	**-0.49**	0.35	0.48	0.28	**-0.65**	0.45
48	The tutor guides students on how to assess their learning progress.	0.18	-0.01	-0.06	**0.68**	0.61	0.36	-0.40	**0.80**
46	The tutor sets individualized learning goals for all students in a small group.	0.14	0.12	0.10	**0.63**	0.50	0.41	-0.24	**0.73**
49	The tutor guides students to generate their own problem lists.	-0.05	0.11	-0.14	**0.58**	0.41	0.38	-0.36	**0.65**

Statistically significant results are marked in bold.

**Table 2.** Correlations between the factors in exploratory and confirmatory factor analyses

	Student-centered	Visual technology	Problem-solving	Self-directed
Correlation (EFA)				
Student-centered	1.00			
Visual technology	0.34	1.00		
Problem-solving	-0.55	-0.28	1.00	
Self-directed	0.59	0.43	-0.36	1.00
Correlation (CFA)				
Student-centered	1.00			
Visual technology	0.42	1.00		
Problem-solving	0.77	0.46	1.00	
Self-directed	0.74	0.55	0.73	1.00

EFA, exploratory factor analysis; CFA, confirmatory factor analysis.

## Supplementary materials

Supplementary files are available from Harvard Dataverse: https://doi.org/10.7910/DVN/Y1VZUK

Supplement 1. Full item list of questionnaire to develop the Student Ratings in Clinical Teaching scale.

Supplement 2. Audio recording of the abstract.
