# Supplementary material for: Development and validation of the student ratings in clinical teaching scale in Australia: a methodological study
Source: J Educ Eval Health Prof. 2023 Sep 5;20:26. doi: 10.3352/jeehp.2023.20.26 (PMC10562831; doi:10.3352/jeehp.2023.20.26)
Supplement: Supplementary file 2 — Supplement 1. Full item list of questionnaire to develop the Student Ratings in Clinical Teaching scale. [file jeehp-20-26-suppl1.pdf]

Anonymous Questionnaire of Teaching Effectiveness

This questionnaire is used to evaluate teaching in your clinical setting. Your response will assist to improve in medical education program.

By completing the anonymous questionnaire, you are providing your consent to the research team to collect and use the unidentifiable information you provided for the research study.

Please think of your most recent clinical learning experience (e.g. rotation) and answer each question by indicating how often you have been taught as described in each of the following items. Place an X in the box which best describes your perception.

Never =1 Always =6

	Never	Rarely	Occasionally	Often	Very Often	Always
1. The tutor utilises technology to illustrate learning.	1	2	3	4	5	6
2. The tutor adds fun activities to the teaching session.	1	2	3	4	5	6
3. The tutor redirects the small group when they go off the task.	1	2	3	4	5	6
4. The tutor encourages students to organise elements in a hierarchical structure.	1	2	3	4	5	6
5. The tutor instructs students how to teach.	1	2	3	4	5	6
6. The tutor guides students to discuss learning processes with peers.	1	2	3	4	5	6
7. The tutor gives personalised instructions to individual students.	1	2	3	4	5	6
8. The tutor encourages students to expand their learning beyond the teaching session.	1	2	3	4	5	6
9. The tutor includes virtual activities in the learning materials.	1	2	3	4	5	6
10. The tutor corrects students in real time when performing a task.	1	2	3	4	5	6
11. The tutor guides students to plan their learning to align with the intended learning outcomes.	1	2	3	4	5	6
12. The tutor encourages students to clarify their levels of understanding.	1	2	3	4	5	6
13. The tutor guides students with methods for considering the pros and cons toward their decisions.	1	2	3	4	5	6
14. The tutor provides students with reports detailing their progress.	1	2	3	4	5	6
15. The tutor rephrases students' questions before answering.	1	2	3	4	5	6
16. The tutor assesses students’ practices based on multiple tasks.	1	2	3	4	5	6
17. The tutor guides students to link ideas in logical ways.	1	2	3	4	5	6
18. The tutor sets clear performance standards for students to meet	1	2	3	4	5	6
19. The tutor encourages students to take responsibility for their learning.	1	2	3	4	5	6

	Never	Rarely	Occasionally	Often	Very Often	Always
20. The tutor assesses individuals' engagement within the group.	1	2	3	4	5	6
21. The tutor guides students about further learning based on their current capabilities.	1	2	3	4	5	6
22. The tutor uses different methods to teach a topic.	1	2	3	4	5	6
23. The tutor guides students to break the learning topic to meaningful propositions.	1	2	3	4	5	6
24. The tutor demonstrates how to apply the knowledge being taught.	1	2	3	4	5	6
25. The tutor provides both individual and group feedback.	1	2	3	4	5	6
26. The tutor encourages students to gather information beneficial to all group members.	1	2	3	4	5	6
27. The tutor guides students to assess their preparedness for the teaching session.	1	2	3	4	5	6
28. The tutor further advances students' competency when their performance is beyond expectation.	1	2	3	4	5	6
29. The tutor encourages students to continue practising after the teaching session.	1	2	3	4	5	6
30. The tutor reduces guidance with improved student performance.	1	2	3	4	5	6
31. The tutor prompts students to split a complex problem into its components.	1	2	3	4	5	6
32. The tutor encourages students to provide peer feedback.	1	2	3	4	5	6
33. The tutor provides students with sufficient time to ask questions.	1	2	3	4	5	6
34. The tutor encourages students to consider a range of hypotheses in problem solving.	1	2	3	4	5	6
35. The tutor acknowledges students’ competent performance.	1	2	3	4	5	6
36. The tutor uses visual technology to enhance understanding of patterns.	1	2	3	4	5	6
37. The tutor guides students to prioritise clinical issues.	1	2	3	4	5	6
38. The tutor designs questions aligned with the relevant learning goals.	1	2	3	4	5	6
39. The tutor blends the teaching materials with creative learning activities.	1	2	3	4	5	6
40. The tutor provides students with guidance for self-assessment.	1	2	3	4	5	6
41. The tutor provides students with real-time feedback.	1	2	3	4	5	6
42. The tutor engages with all students in a small group teaching session.	1	2	3	4	5	6
43. The tutor uses quizzes in the teaching sessions.	1	2	3	4	5	6
44. The tutor guides students on how to visualise abstract concepts.	1	2	3	4	5	6
45. The tutor prompts students to clarify their reasoning.	1	2	3	4	5	6

	Never	Rarely	Occasionally	Often	Very Often	Always
46. The tutor sets individualised learning goals for all students in a small group.	1	2	3	4	5	6
47. The tutor demonstrates how to solve problems step by step.	1	2	3	4	5	6
48. The tutor guides students on how to assess their learning progress.	1	2	3	4	5	6
49. The tutor guides students to generate their own problem lists.	1	2	3	4	5	6
50. The tutor provides clear explanations as to what needs to be improved on their skills or knowledges.	1	2	3	4	5	6
51. The tutor provides students feedback on how to complete the assigned task more efficiently.	1	2	3	4	5	6
52. The tutor encourages students to ask questions.	1	2	3	4	5	6
53. The tutor monitors the intergroup members' behaviours.	1	2	3	4	5	6
54. The tutor encourages students to share their learning goals with other students.	1	2	3	4	5	6
55. The tutor guides students to draw figures illustrating processes.	1	2	3	4	5	6
56. The tutor uses summative assessments during the teaching session.	1	2	3	4	5	6
57. The tutor asks students to demonstrate the steps of the solution to the problem.	1	2	3	4	5	6
58. The tutor encourages disengaged students to contribute ideas to the group.	1	2	3	4	5	6
59. The tutor uses technology to facilitates visual feedback to students.	1	2	3	4	5	6
60. The tutor guides students to demonstrate skills to their peers.	1	2	3	4	5	6
61. The tutor facilitates students to practice interpersonal communication skills with peers.	1	2	3	4	5	6
62. The tutor addresses the gaps between individual students’ current performance and the intended learning outcomes.	1	2	3	4	5	6
63. The tutor requires students to include illustrations in their reports	1	2	3	4	5	6
64. The tutor guides students to practice similar skills across different situations.	1	2	3	4	5	6
65. The tutor encourages students to focus on a shared learning goal.	1	2	3	4	5	6
66. The tutor adapts their practice to address students' feedback on their teaching.	1	2	3	4	5	6
67. The tutor helps students to increase their performance accurately.	1	2	3	4	5	6
68. The tutor guides students to reflect on their own learning.	1	2	3	4	5	6
69. The tutor facilitates students to practice differential diagnosis.	1	2	3	4	5	6
70. The tutor places students in heterogenous interactive learning groups.	1	2	3	4	5	6

	Never	Rarely	Occasional ly	Often	Very Often	Always
71. The tutor encourages students to visualise abstract concepts.	1	2	3	4	5	6
72. The tutor links the teaching materials to the intended learning outcomes.	1	2	3	4	5	6
73. The tutor acknowledges students accomplishment during the teaching session.	1	2	3	4	5	6
74. The tutor challenges students' current knowledge.	1	2	3	4	5	6
75. The tutor facilitates students to learn from observing peers.	1	2	3	4	5	6
76. The tutor explains the intended learning outcomes to students.	1	2	3	4	5	6
77. The tutor encourages students to systematically gather information.	1	2	3	4	5	6
78. The tutor discusses with students about future challenges in their study.	1	2	3	4	5	6

Please let us know more about yourself:

Gender: ☐ Male ☐ Female

Age: ________

Year of study: ☐ Year 2/ ☐ Year 3/ ☐ Year 6

Most recent clinical learning experience: ______________________
(i.e. Cardiology, Plastic surgery, Paediatrics…)

Is English your first language? ☐ Yes / ☐ No

Status: ☐ Domestic / ☐ International

Thank you very much for your time and cooperation.
